# Carbon:Nitrogen:Phosphorus Stoichiometry in Fungi: A Meta-Analysis

**DOI:** 10.3389/fmicb.2017.01281

**Published:** 2017-07-14

**Authors:** Ji Zhang, James J. Elser

**Affiliations:** ^1^Institute of Medicinal Plants, Yunnan Academy of Agricultural Sciences Kunming, China; ^2^School of Life Sciences, Arizona State University, Tempe AZ, United States; ^3^Flathead Lake Biological Station, University of Montana, Polson MT, United States

**Keywords:** elemental composition, fungus, guild, homeostasis, Redfield ratios, stoichiometry

## Abstract

Surveys of carbon:nitrogen:phosphorus ratios are available now for major groups of biota and for various aquatic and terrestrial biomes. However, while fungi play an important role in nutrient cycling in ecosystems, relatively little is known about their C:N:P stoichiometry and how it varies across taxonomic groups, functional guilds, and environmental conditions. Here we present the first systematic compilation of C:N:P data for fungi including four phyla (Ascomycota, Basidiomycota, Glomeromycota, and Zygomycota). The C, N, and P contents (percent of dry mass) of fungal biomass varied from 38 to 57%, 0.23 to 15%, and 0.040 to 5.5%, respectively. Median C:N:P stoichiometry for fungi was 250:16:1 (molar), remarkably similar to the canonical Redfield values. However, we found extremely broad variation in fungal C:N:P ratios around the central tendencies in C:N:P ratios. Lower C:P and N:P ratios were found in Ascomycota fungi than in Basidiomycota fungi while significantly lower C:N ratios (*p* < 0.05) and higher N:P ratios (*p* < 0.01) were found in ectomycorrhizal fungi than in saprotrophs. Furthermore, several fungal stoichiometric ratios were strongly correlated with geographic and abiotic environmental factors, especially latitude, precipitation, and temperature. The results have implications for understanding the roles that fungi play in function in symbioses and in soil nutrient cycling. Further work is needed on the effects of actual *in situ* growth conditions of fungal growth on stoichiometry in the mycelium.

## Introduction

Ecological stoichiometry is the study of the balance of multiple chemical elements in ecological interactions and processes (Sterner and Elser, 2002). By using the perspective of ecological stoichiometry, we can better understand the coupling of energy and material flows at key interfaces in Earth’s diverse habitats (Elser et al., 2000a). For example, the features of the stoichiometry of algae–zooplankton interactions have been shown to affect trophic transfer efficiencies and consumer-driven nutrient recycling at the ecosystem scale (Frost et al., 2002; Andersen et al., 2004). Moreover, the extension of ecological stoichiometry to biological phenomena, referred to as biological stoichiometry (Elser et al., 2000c), provides a mechanistic theory linking cellular and biochemical features of co-evolving biota with constraints imposed by, and impacts on, ecosystem energy and nutrient flows (Elser et al., 2003).

To date, studies involving ecological stoichiometry and biological stoichiometry have mostly been done in aquatic systems (McGroddy et al., 2004), but recent work has extended it to new research areas beyond the aquatic realm (Hessen et al., 2013) such as study of terrestrial vegetation (Kerkhoff et al., 2006), insects (Woods et al., 2004), and soils (Cleveland and Liptzin, 2007). As nutrient cycling in natural ecosystems is largely driven by microorganisms (Spohn, 2016), the carbon:nitrogen:phosphorus ratios of soil microbial biomass and ecoenzymatic activity ratios related to resource acquisition have begun to be surveyed (Cleveland and Liptzin, 2007; Sinsabaugh et al., 2009), with a primary emphasis on bacteria. Differences in fungal and bacterial physiology may have important influences on large scale C and N cycling (Waring et al., 2013). However, relatively little is known about fungal stoichiometry despite the important role they play in biogeochemical cycling in ecosystems (Gadd, 2004, 2007). It is still not clear that how these processes might be influenced by the C:N:P stoichiometric requirements of the fungi themselves. In one of the first studies of fungal C:N:P stoichiometry, Danger et al. (2016) assessed the ecological stoichiometry of aquatic fungi and several species of terrestrial fungi, finding that the variation of C:N:P ratios of fungal biomass exceeded variations found for bacteria. However, fungal biodiversity is considerably higher in terrestrial than in aquatic ecosystems (Bärlocher and Boddy, 2016) but our knowledge of their stoichiometry remain lacking.

The role of fungi in symbiosis, e.g., ectomycorrhizae, is of particular interest. Most plant species form symbioses with soil fungi, and up to 80% of plant N and P is provided by mycorrhizal fungi (Behie and Bidochka, 2014; van der Heijden et al., 2015). Mycorrhizal fungi can use organic nitrogen and phosphorus forms, which would otherwise remain largely unavailable to plant roots (Landeweert et al., 2001). Intriguingly, the reciprocity of benefits to host plant and fungus are highly dependent upon the C:P stoichiometry of the transaction (Schwartz and Hoeksema, 1998). Since mycorrhizal fungi explore the soil volume for nutrients as part of this symbiotic transaction, it is important to better understand the resource requirements of fungi symbiosis. In particular, what are N and P requirements of the fungal partner? Answering such simple questions is difficult because our understanding of fungal C, N, and P stoichiometry and how it varies across taxonomic groups, functional guilds, and environmental conditions is poorly developed.

To address these gaps in our knowledge, here we present the first systematic compilation of C:N:P data for fungi. Our questions are as follows. What range and average values of C:N:P ratios are exhibited by fungi? Does fungal stoichiometry differ among phyla or functional guilds? Do environmental factors affect fungal stoichiometry? Answering these questions will allow stoichiometric theory to be brought to bear on key ecological processes driven by fungi.

## Materials and Methods

We performed a systematic literature search in ISI Web of Science and Google Scholar using combinations of key words including stoichiometry, element, carbon, nitrogen, phosphorus, chemical composition, mineral, nutrient, fungi, mushroom, yeast, Saccharomyces, fruit body, sporocarp, mycorrhizal, mycelium, and hyphae. We also followed cited references in the identified literature to find additional relevant studies.

Contents (percent of dry mass) or ratios of C, N, and P were extracted from published studies, either from tables or from figures by WebPlotDigitizer^1^. Studies that directly reported either absolute protein and RNA (or rRNA) content or protein:RNA ratio were also selected. In this case, studies that measured macromolecular content only under severe limitation and far from optimal growth conditions were excluded (Loladze and Elser, 2011). The protein:RNA ratio was converted to N:P ratio (28 entries for 18 species) according to a model developed by Loladze and Elser (2011). For studies that did not report %C, we used the average of C content (%C) data from the literature (44.0) in order to calculate C:N or C:P ratios from N content and P content values. Records without detailed taxonomic information (e.g., unidentified fungi) were excluded. See Supplementary Materials for the full dataset and references (Appendix S1, S2).

Fungal nomenclature followed the Index Fungorum^2^. Information for 377 fungi species (101 genera from 82 families) was assembled across a broad range of diversity including four phyla (Ascomycota, Basidiomycota, Glomeromycota, and Zygomycota). We used Funguild, a new open annotation tool, to assign the fungal species to the functional guilds (Nguyen et al., 2016). Ectomycorrhizal fungi, pathotrophs (receiving nutrients by harming host cells), and saprotrophs were selected for comparison of C:N:P stoichiometry among functional guilds (Appendix S3).

To assess possible correlations of fungal C:N:P ratios with climatic conditions, mean annual temperature, and mean annual precipitation data from 1950 to 2000 for the sampling sites of Agaricomycetes species were obtained from the WorldClim database^3^ at a spatial resolution of 30 arc-seconds (ca.1 km) using Diva-GIS 7.5 software^4^ (Hijmans et al., 2005). Agaricomycetes were the taxonomic group for which we had the most data, providing sufficient basis for assessment of climatic and geographic factors. Most of the element data for the correlation analysis are from fruiting bodies due to the lack of data for mycelia.

Elemental concentration and stoichiometric ratio data (%C, %N, %P, C:N, C:P, and, N:P) were log10-transformed before analyses to improve the normality of residuals. Student’s *t*-test was used to compare the concentration and ratios in fungus species for two phyla, Ascomycota and Basidiomycota, for which we had a large number of observations. ANOVA followed by Tukey’s *post hoc* test were used to assess the statistical significance of differences in the variables among different functional guilds. Pearson’s correlation analysis was performed to check for correlations between fungal C:N:P ratios and various geographic and abiotic environmental factors. All analyses were performed using R 3.1.3 program (R Core Team, 2015).

## Results

**FIGURE 1:**
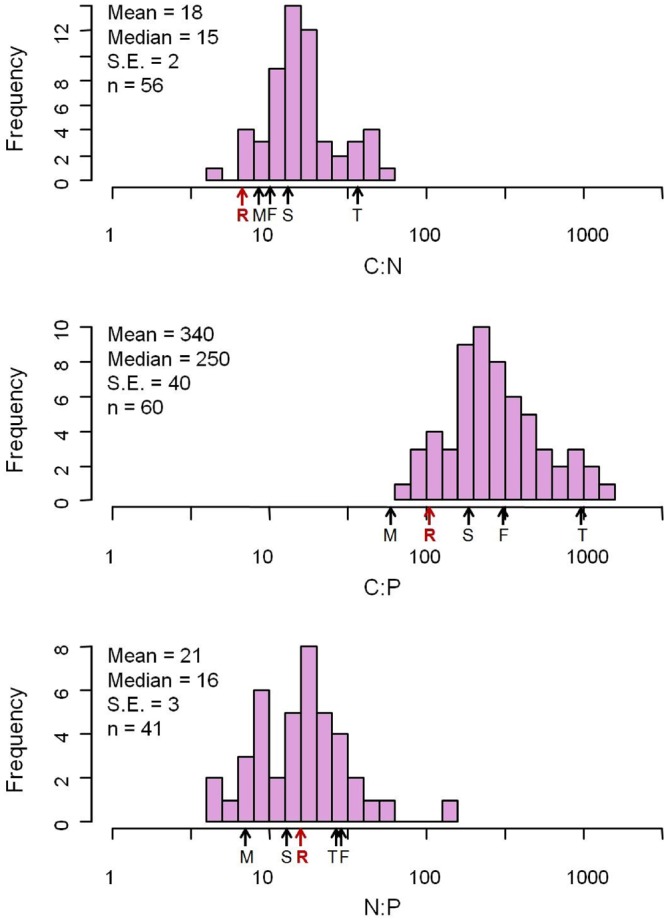
Distribution of C:N:P molar ratios from 377 fungus species. The mean C:N:P ratios for major biota and ecosystems from previous studies are marked with arrows. F, freshwater autotrophs (Elser et al., 2000a); M, soil microbial biomass (Cleveland and Liptzin, 2007); R, Redfield (Redfield, 1958); T, terrestrial autotrophs (Elser et al., 2000a); S, soil (Cleveland and Liptzin, 2007).

The elemental concentrations of fungal biomass varied considerably: from 38 to 57% for %C, 0.23 to 15% for %N, and 0.040 to 5.5% for %P (Appendix S2). The median C:N:P ratio was 250:16:1 (molar, **Figure 1** and **Table 1**), a value with N:P ratio remarkably close to the canonical Redfield ratio of 16:1 (Redfield, 1958).

**Table 1:** Contents and ratios of C, N, and P in fungi at different taxonomic levels.

Taxonomic level		%C	%N	%P	C:N	C:P	N:P
Species	Mean	43.75	3.95	0.61	17.23	322.44	19.64
	Median	43.40	3.70	0.53	13.65	222.88	15.06
	Minimum	38.19	0.25	0.05	3.98	47.56	2.80
	Maximum	57.10	12.90	2.39	203.95	2085.63	141.71
	Number	128	238	232	252	246	105
	*SD*	2.32	1.74	0.39	16.75	303.97	18.48
Genus	Mean	44.08	3.96	0.59	18.71	327.13	19.74
	Median	43.88	3.73	0.52	14.13	242.41	15.83
	Minimum	38.80	0.52	0.06	4.50	76.70	2.80
	Maximum	57.10	11.40	1.76	126.19	1854.72	141.71
	Number	62	97	116	110	128	70
	*SD*	2.88	2.00	0.34	15.96	265.55	17.98
Family	Mean	44.04	3.66	0.57	18.47	344.68	21.21
	Median	44.09	3.57	0.52	15.16	249.74	16.05
	Minimum	39.73	0.97	0.10	4.55	76.70	4.39
	Maximum	50.80	8.18	1.55	52.92	1284.01	141.71
	Number	37	48	54	56	60	41
	*SD*	2.32	1.56	0.31	11.31	271.69	22.24

**FIGURE 2:**
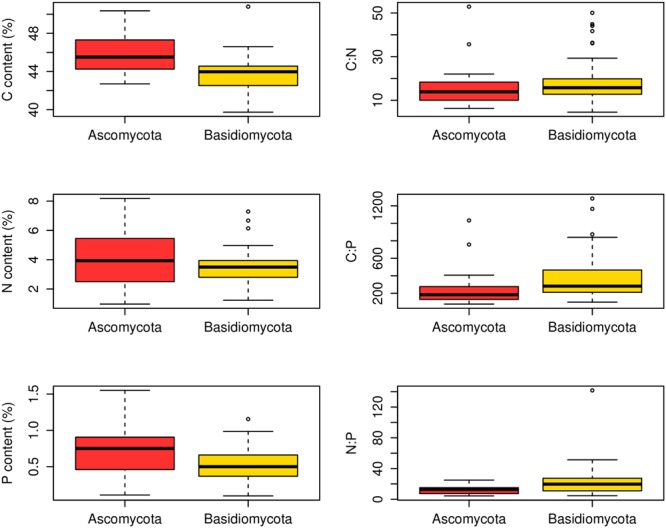
Contents and ratios of fungi C, N, and P between different phyla. Significant differences (*p* < 0.05) between Ascomycota (23 families) and Basidiomycota (49 families) fungi were found in C content and C:P and N:P ratios.

**FIGURE 3:**
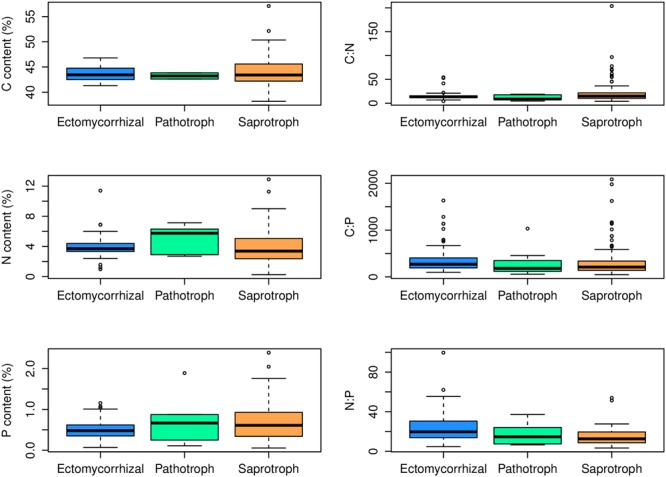
Contents and ratios of fungi C, N, and P among different functional guilds. Significant lower C:N ratio (*p* < 0.05) and higher N:P ratio (*p* < 0.01) were found in the ectomycorrhizal fungi rather than in saprotrophs.

At the family level, significant differences (*p* < 0.05) between Ascomycota and Basidiomycota fungi in C content and C:P and N:P ratios were found (**Figure 2**). Among the three fungal guilds considered, significantly lower C:N ratio (*p* < 0.05) and higher N:P ratio (*p* < 0.01) were observed in ectomycorrhizal species compared to saprotrophic species (**Figure 3** and **Table 2**).

**Table 2:** Contents and ratios of fungal C, N, and P among different functional guilds.

Functional guild		%C	%N	%P	C:N	C:P	N:P
Ectomycorrhizal fungi	Mean	43.67	3.89	0.58	14.44	348.30	24.61
	Median	43.45	3.70	0.48	13.65	270.13	19.75
	Minimum	41.30	0.97	0.07	4.50	10.63	4.58
	Maximum	46.80	11.40	4.60	54.33	1633.14	99.64
	Number	84	135	90	135	91	37
	*SD*	1.41	1.10	0.64	6.08	271.92	18.28
Pathotroph	Mean	42.60	4.41	6.90	13.04	317.77	11.92
	Median	42.60	4.33	0.87	13.09	195.46	7.38
	Minimum	42.60	2.70	0.11	7.13	58.37	6.39
	Maximum	42.60	6.30	43.85	19.01	1033.33	24.06
	Number	1	4	7	6	7	5
	*SD*		1.88	16.30	5.63	341.38	7.55
Saprotroph	Mean	43.86	5.66	1.28	21.19	312.04	55.69
	Median	43.30	3.29	0.65	14.16	192.27	13.12
	Minimum	38.19	0.25	0.055	0.30	1.17	3.24
	Maximum	57.10	47.40	42.30	203.95	2085.63	473.33
	Number	28	71	102	86	112	62
	*SD*	3.83	9.01	4.22	27.02	360.57	106.69

N:P ratios of Agaricomycete fungi increased toward the equator, coincident with increases in average temperature and precipitation (**Table 3**). However, elemental contents and stoichiometric ratios in this group showed little direct correlation with elevation (**Table 3**).

**Table 3:** Pearson’s correlation coefficients for the Agaricomycete fungi C, N, and P contents and ratios and four abiotic environmental variables.

Trait	Absolute latitude	Elevation	Mean annual precipitation	Mean annual temperature
C	–0.174ˆ**	–0.089	–0.125ˆ*	0.147ˆ**
N	–0.075	–0.025	–0.133ˆ*	0.144ˆ**
P	–0.062	–0.006ˆ*	–0.244ˆ**	–0.075
C:N	–0.099	–0.020	–0.244ˆ**	0.025
C:P	–0.071	–0.143ˆ*	–0.101	0.229ˆ**
N:P	–0.246ˆ**	–0.108	–0.191ˆ*	0.341ˆ**

*^∗^*p* < 0.05, ^∗∗^*p* < 0.01.*

## Discussion

### Large-Scale Patterns of Fungal N:P

Our data reveal that elemental ratios of fungal biomass vary from 3.3 to 220 (C:N), 21 to 2800 (C:P), and 1.5 to 140 (N:P) (Appendix S2). The variation of these ratios is considerable and exceeds that reported for fungi, bacteria or whole microbial communities in previous studies (Cleveland and Liptzin, 2007; Mouginot et al., 2014; Danger et al., 2016), although the highest N:P ratio we report is less than the extremely high N:P ratio (400) of microbes from hot springs of Yellowstone National Park (Neveu et al., 2016). Overall, however, our data for fungi bear remarkable resemblance to stoichiometric properties of other taxa. Most notable is that the median N:P ratio of fungal biomass was 16:1, identical to the canonical Redfield ratio for marine phytoplankton (Redfield, 1958).

This remarkable outcome highlights the influence of a strong central tendency in N:P ratio due to the core biochemical investments associated with N-rich proteins and P-rich protein synthesis machinery (e.g., ribosomal RNA) that holds across all biota (Loladze and Elser, 2011). However, there was extremely broad variation in C:N:P ratios around this central value, variation that likely reflects both local biochemical variation due to physiological adjustment to environmental conditions (Klausmeier et al., 2004) but also broader phylogenetic influences of different fungal taxa exhibiting different life history strategies in particular environments.

The poleward decline of N:P in our data (**Table 3**) also bears a striking similarity to patterns seen for other biota and ecosystems. For example, leaf N:P ratios decline with latitude (McGroddy et al., 2004; Reich and Oleksyn, 2004; Kerkhoff et al., 2005) as do N:P ratios in multicellular and unicellular photoautotrophs in freshwater and marine ecosystems (Borer et al., 2013; Martiny et al., 2013). Elser et al. (2000b) reported a similar poleward increase in P demands in the crustacean *Daphnia*, invoking selection for rapid growth (and thus P-rich RNA) due to the short growing season as an explanation. Decreases in soil microbial N:P ratios on the Tibetan Plateau as a function of latitude were reported by (Chen et al., 2016) and were associated with changes in soil microbial community structures. However, Cleveland and Liptzin (2007) found that microbial N:P ratios in soil microbial biomass were constrained at the global scale and did not show a latitudinal pattern.

Foliar nutrient contents tend to increase with altitude (Körner, 1989). In contrast to studies of foliar N:P ratios in plants along mountain slopes (Morecroft and Woodward, 1996; van de Weg et al., 2009), we found no correlation of fungal N:P ratios with elevation. This may have been the result of a confounding effect driven by the negative relationship between absolute latitude and elevation of the sampling sites in our dataset (Appendix S2).

In general, foliar N:P ratios increase with temperature across large geographic gradients (Reich and Oleksyn, 2004). We found a similar trend in fungal N:P ratio (**Table 3**). However, while temperature explained a substantial proportion of the total variability in leaf P (Reich and Oleksyn, 2004), variation in fungal P showed no detectable association with temperature. This may be due, in part, to the smaller temperature range from 1.5°C to 26.6°C in our data set compared with the -12.8°C to 28.0°C range considered by Reich and Oleksyn (2004). As most of the element data for the correlation analysis in our study are from fruiting bodies, further work is needed on the effects of actual *in situ* growth conditions (temperature and humidity) of fungal growth on stoichiometry in the mycelium.

While fungal N:P ratios changed with environmental conditions, it is unclear if the patterns we observe reflect shifts in the species present (with each species stoichiometrically homeostatic around a particular value) or if the ratios might vary if a given species was grown across the full range of environmental (soil, temperature, N, P, etc) conditions. This issue requires further investigation. How resource stoichiometry affects fungi is of particular interest (Danger et al., 2016) but data on nutrient availability are found in only a few studies. For instance, in terrestrial fungi, phylogenetically related strains can have distinct stoichiometric ratios despite the same growth conditions (Mouginot et al., 2014). In aquatic fungi, Leach and Gulis (2010) reported that fungal biomass is homeostatic with respect to C:N and N:P but is weakly homeostatic with respect to C:P. However, Danger and Chauvet (2013) found highly plastic C:P and N:P ratios in aquatic hyphomycetes as a function of external nutrient supply.

### Stoichiometry in Mycorrhizal Symbioses

While the central similarity of average fungal C:N:P stoichiometry to previous observations is notable, the differences of N:P among fungal phyla that we observed needs further investigation. At least some of this variation may be associated with different functional guilds within fungal groups (Talbot et al., 2015). For example, in this study, ectomycorrhizal fungi had lower C:N ratio and higher N:P ratio compared with saprotrophs, supporting a role for trophic guild in influencing fungal C:N:P stoichiometry. The ectomycorrhizal fungal lifestyle has evolved multiple times from saprotrophic lineages of wood and litter decomposers through convergent evolution (van der Heijden et al., 2015). Contrasting effects of nitrogen availability on plant carbon supply to mycorrhizal fungi and saprotrophs may exist (Högberg et al., 2003). A considerable amount of research has been aimed at assessing the ability of ectomycorrhizal fungi to use organic nitrogen sources (Chalot and Brun, 1998). Ectomycorrhizal fungi benefit from organic matter decomposition primarily through increased nitrogen mobilization (Lindahl and Tunlid, 2015). Compared to saprotrophs, mycorrhizal fungi have a stable supply of C from their hosts, so they may exploit organic substrates selectively for N and other nutrients (Bödeker et al., 2016). A recent study has shown that ectomycorrhizal species have lower N content than saprotrophic fungal species (Trocha et al., 2016). However, we found that the N content of saprotrophic fungal species had a wider range than ectomycorrhizal species but the average N content did not differ significantly between the two guilds. We suggest that additional information about the C:N:P stoichiometry of functional groups of fungi may shed considerable light on the nature of various symbioses as well as on the functioning of various fungi as a reflection of their functional group.

### Implications for Soil Nutrient Cycling

Soil microbial communities strongly affect element cycling at the ecosystem scale by adjusting the rates of various element acquisition processes (organic matter decomposition, N_2_ fixation, and P solubilization) to acquire limiting resources or by adjusting partitioning and turnover times in the microbial biomass (Spohn, 2016). For instance, global nitrogen-release patterns can be explained by fundamental stoichiometric relationships of decomposer activity (Manzoni et al., 2008). Heterotrophic microbial communities, which drive much of the nutrient cycling in soils, have received increasing interest in recent years (Zechmeister-Boltenstern et al., 2015). Indeed, Cleveland and Liptzin (2007) suggested that an average soil microbial N:P ratio might be a more appropriate index of ecosystem nutrient limitation than plant N:P ratios. A recent study showed that the biogeochemical consequences of N deposition in temperate forests may be driven by the stoichiometry of the dominant trees and their associated microbes (Midgley and Phillips, 2016) while van Diepen et al. (2017) found that fungi exposed to chronic nitrogen enrichment are less able to decay leaf litter. All of these studies point to the importance of multi-resource interactions in nutrient cycling in soils. To advance our understanding of how fungi contribute to these processes, further studies are needed of the relationship between fungal and soil C:N:P ratios as a function of nutrient supply, temperature, and precipitation in order to establish the strength of C:N:P stoichiometric homeostasis in soil fungi. This information will help in many areas of terrestrial ecosystem ecology, including in improving our models of the impacts of fungi on N and P immobilization vs. mineralization.

Our analysis indicates extremely broad variation in fungal C:N:P ratios around a core central tendency in N:P ratio that is notably similar to observations for other biota. Our analyses suggest that variation in fungal C:N:P stoichiometry likely reflects both local biochemical variation due to physiological adjustment to environmental conditions but also broader phylogenetic influences of different fungal guilds in particular environments.

## Author Contributions

JZ and JE conceived and designed the study; JZ analyzed the data; JZ and JE wrote the paper.

## Conflict of Interest Statement

The authors declare that the research was conducted in the absence of any commercial or financial relationships that could be construed as a potential conflict of interest.

## Footnotes

1 http://arohatgi.info/WebPlotDigitizer/

2 http://www.indexfungorum.org

3 http://worldclim.org/current

4 http://www.diva-gis.org
