# Supplementary material for: Carbon:Nitrogen:Phosphorus Stoichiometry in Fungi: A Meta-Analysis
Source: Front Microbiol. 2017 Jul 14;8:1281. doi: 10.3389/fmicb.2017.01281 (PMC5508194; doi:10.3389/fmicb.2017.01281)
Supplement: Supplementary file 1 [file Data_Sheet_1.DOCX]

Supplementary Material

Carbon: Nitrogen: Phosphorus Stoichiometry in Fungi: A Meta-Analysis

Ji Zhang, James J. Elser*

*** Correspondence:** Dr. James J. Elser: jim.elser@flbs.umt.edu

# Appendix S1 References from which the data were obtained.

Data used for meta-analysis were collected from the articles listed below.

Aguilar Uscanga, M.G., Délia, M.L., and Strehaiano, P. (2003). Brettanomyces bruxellensis: effect of oxygen on growth and acetic acid production. *Appl. Microbiol. Biot.* 61, 157-162. doi: 10.1007/s00253-002-1197-z.

Alberghina, F., Sturani, E., and Gohlke, J.R. (1975). Levels and rates of synthesis of ribosomal ribonucleic acid, transfer ribonucleic acid, and protein in *Neurospora crassa* in different steady states of growth. *J. Biol. Chem.* 250, 4381-4388.

Allen, M.F., Swenson, W., Querejeta, J.I., Egerton-Warburton, L.M., and Treseder, K.K. (2003). Ecology of mycorrhizae: A conceptual framework for complex interactions among plants and fungi. *Annu. Rev. Phytopathol.* 41, 271-303. doi: 10.1146/annurev.phyto.41.052002.095518.

Arce, S., Cerutti, S., Olsina, R., Gomez, M.R., and Martnez, L.D. (2008). Trace element profile of a wild edible mushroom (*Suillus granulatus*). *J. Aoac Int.* 91, 853-857.

Ayaz, F.A., Torun, H., Colak, A., Sesli, E., Millson, M., and Glew, R.H. (2011). Macro- and microelement contents of fruiting bodies of wild-edible mushrooms growing in the East Black Sea Region of Turkey. *Food and Nutrition Sciences* 2, 53-59. doi: 10.4236/fns.2011.22007.

Barford, J.P., and Hall, R.J. (1979). Investigation of the significance of a carbon and redox balance to the measurement of gaseous metabolism of *Saccharomyces cerevisiae*. *Biotechnol. Bioeng.* 21, 609-626. doi: 10.1002/bit.260210407.

Boehlke, K.W., and Friesen, J.D. (1975). Cellular content of ribonucleic acid and protein in *Saccharomyces cerevisiae* as a function of exponential growth rate: calculation of the apparent peptide chain elongation rate. *J. Bacteriol.* 121, 429-33.

Brookes, P.C., Powlson, D.S., and Jenkinson, D.S. (1982). Measurement of microbial biomass phosphorus in soil. *Soil Biol. Biochem.* 14, 319-329. doi: 10.1016/0038-0717(82)90001-3.

Brown, C.M., and Rose, A.H. (1969). Effects of temperature on composition and cell volume of *Candida utilis*. *J. Bacteriol.* 97, 261.

Bučinová, K., Janík, R., Jamnická, G., and Kuklová, M. (2014). Accumulation and bioconcentration factors of mineral macronutrients in representative species of macrofungi prevailing in beech-dominated forests affected by air pollution. *Czech Mycology* 66, 193-207.

Campos, J.A. (2011). Nutrients and Trace Elements Content of Wood Decay Fungi Isolated from Oak (*Quercus ilex*). *Biol. Trace Elem. Res.* 144, 1370-1380. doi: 10.1007/s12011-011-9134-6.

Carnicer, M., Baumann, K., Toplitz, I., Sanchez-Ferrando, F., Mattanovich, D., Ferrer, P., and Albiol, J. (2009). Macromolecular and elemental composition analysis and extracellular metabolite balances of Pichia pastoris growing at different oxygen levels. *Microb. Cell Fact.* 8, 65. doi: 10.1186/1475-2859-8-65.

Chojnacka, A., Jarzynska, G., Lewandowska, M., Nnorom, I.C., and Falandysz, J. (2013). Trace elements of Yellow-Cracking Bolete [*Xerocomus subtomentosus* (l.) Quél.] collected at the same site over three years. *Fresen. Environ. Bull.* 22, 2707-2712.

Chudzynski, K., Jarzynska, G., and Falandysz, J. (2013). Cadmium, lead and some other trace elements in Larch Bolete mushrooms (*Suillus grevillei*) (Klotzsch) Sing., collected from the same site over two years. *Food additives & contaminants. Part B, Surveillance* 6, 249-53. doi: 10.1080/19393210.2013.807881.

Chudzyński, K., and Falandysz, J. (2008). Multivariate analysis of elements content of Larch Bolete (*Suillus grevillei*) mushroom. *Chemosphere* 73, 1230-1239. doi: 10.1016/j.chemosphere.2008.07.055.

Clinton, P.W., Buchanan, P.K., and Allen, R.B. (1999). Nutrient composition of epigeous fungal sporocarps growing on different substrates in a New Zealand mountain beech forest. *New Zeal. J. Bot.* 37, 149-153.

Cohen, N., Cohen, J., Asatiani, M.D., Varshney, V.K., Yu, H.T., Yang, Y.C., Li, Y.H., Mau, J.L., and Wasser, S.P. (2014). Chemical Composition and Nutritional and Medicinal Value of Fruit Bodies and Submerged Cultured Mycelia of Culinary-Medicinal Higher Basidiomycetes Mushrooms. *Int. J. Med. Mushrooms* 16, 273-291.

Cvetkovic, J.S., Mitic, V.D., Stankov-Jovanovic, V.P., Dimitrijevic, M.V., and Nikolic-Mandic, S.D. (2015). Elemental composition of wild edible mushrooms from Serbia. *Anal. Lett.* 48, 2107-2121.

Danger, M., and Chauvet, E. (2013). Elemental composition and degree of homeostasis of fungi: Are aquatic hyphomycetes more like metazoans, bacteria or plants? *Fungal Ecol.* 6, 453-457. doi: 10.1016/j.funeco.2013.05.007.

Dekkers, J., Dekok, H.E., and Roels, J.A. (1981). Energetics of Saccharomyces cerevisiae CBS 426: Comparison of anaerobic and aerobic glucose limitation. *Biotechnol. Bioeng.* 23, 1023-1035. doi: 10.1002/bit.260230510.

Dimitrijevic, M.V., Mitic, V.D., Cvetkovic, J.S., Stankov Jovanovic, V.P., Mutic, J.J., and Nikolic Mandic, S.D. (2016). Update on element content profiles in eleven wild edible mushrooms from family Boletaceae. *Eur. Food Res. Technol.* 242, 1-10. doi: 10.1007/s00217-015-2512-0.

Dostalek, P., Patzak, M., and Matejka, P. (2004). Influence of specific growth limitation on biosorption of heavy metals by Saccharomyces cerevisiae. *Int. Biodeter. Biodegr.* 54, 203-207. doi: 10.1016/j.ibiod.2004.03.013.

Drewnowska, M., and Falandysz, J. (2015). Investigation on mineral composition and accumulation by popular edible mushroom common chanterelle (Cantharellus cibarius). *Ecotox. Environ. Safe.* 113, 9-17. doi: 10.1016/j.ecoenv.2014.11.028.

Duboc, P., Schill, N., Menoud, L., van Gulik, W., and von Stockar, U. (1995). Measurements of sulfur, phosphorus and other ions in microbial biomass: influence on correct determination of elemental composition and degree of reduction. *J. Biotechnol.* 43, 145-58.

Edmonds, R.L., and Lebo, D.S. (1998). Diversity, production, and nutrient dynamics of fungal sporocarps on logs in an old-growth temperate rain forest, Olympic National Park, Washington. *Canadian Journal of Forest Research* 28, 665-673. doi: 10.1139/cjfr-28-5-665.

Egli, T.H., and Quayle, J.R. (1986). Influence of the carbon: nitrogen ratio of the growth medium on the cellular composition and the ability of the methylotrophic yeast *Hansenula polymorpha* to utilize mixed carbon sources. *Journal of General Microbiology* 132, 1779-1788.

Falandysz, J., Chudzynski, K., Kojta, A.K., Jarzynska, G., and Drewnowska, M. (2012). Comparison of two acid extraction methods for determination of minerals in soils beneath to Larch Bolete (*Suillus grevillei*) and aimed to estimate minerals sequestration potential in fruiting bodies. *Journal of Environmental Science and Health, Part A* 47, 1607-13. doi: 10.1080/10934529.2012.680781.

Falandysz, J., Drewnowska, M., Jarzyńska, G., Zhang, D., Zhang, Y., and Wang, J. (2012). Mineral constituents in common chanterelles and soils collected from a high mountain and lowland sites in Poland. *J. Mt. Sci.-Engl.* 9, 697-705. doi: 10.1007/s11629-012-2381-y.

Falandysz, J., Kunito, T., Kubota, R., Bielawski, L., Frankowska, A., Falandysz, J.J., and Tanabe, S. (2008). Multivariate characterization of elements accumulated in King Bolete *Boletus edulis* mushroom at lowland and high mountain regions. *Journal of Environmental Science and Health, Part A* 43, 1692-9. doi: 10.1080/10934520802330206.

Falandysz, J., Szymczyk, K., Ichihashi, H., Bielawski, L., Gucia, M., Frankowska, A., and Yamasaki, S.I. (2001). ICP/MS and ICP/AES elemental analysis (38 elements) of edible wild mushrooms growing in Poland. *Food Additives and Contaminants* 18, 503-513. doi: 10.1080/0265203001002762.

Furukawa, K., Heinzle, E., and Dunn, I.J. (1983). Influence of oxygen on the growth of *Saccharomyces cerevisiae* in continuous culture. *Biotechnol. Bioeng.* 25, 2293-2317. doi: 10.1002/bit.260251003.

Gençcelep, H., Uzun, Y., Tunçtürk, Y., and Demirel, K. (2009). Determination of mineral contents of wild-grown edible mushrooms. *Food Chem.* 113, 1033-1036. doi: 10.1016/j.foodchem.2008.08.058.

George, P.L., Ranatunga, T.D., Reddy, S.S., and Sharma, G.C. (2014). A comparative analysis of mineral elements in the mycelia and the fruiting bodies of Shiitake mushrooms. *American Journal of Food Technology* 9, 360-369.

Grimmett, I.J., Shipp, K.N., Macneil, A., and Bärlocher, F. (2013). Does the growth rate hypothesis apply to aquatic hyphomycetes? *Fungal Ecol.* 6, 493-500. doi: 10.1016/j.funeco.2013.08.002.

Gucia, M., Jarzynska, G., Kojta, A.K., and Falandysz, J. (2012). Temporal variability in 20 chemical elements content of Parasol Mushroom (*Macrolepiota procera*) collected from two sites over a few years. *Journal of Environmental Science and Health, Part B* 47, 81-8. doi: 10.1080/03601234.2012.611433.

Gucia, M., Jarzyńska, G., Rafał, E., Roszak, M., Kojta, A.K., Osiej, I., and Falandysz, J. (2012). Multivariate analysis of mineral constituents of edible Parasol Mushroom *(Macrolepiota procera*) and soils beneath fruiting bodies collected from Northern Poland. *Environ. Sci. Pollut. R.* 19, 416-431. doi: 10.1007/s11356-011-0574-5.

Guo, L., Lin, J., and Lin, J. (2007). Non-volatile components of several novel species of edible fungi in China. *Food Chem.* 100, 643-649. doi: 10.1016/j.foodchem.2005.09.087.

Györfi, J., Geösel, A., and Vetter, J. (2010). Mineral composition of different strains of edible medicinal mushroom *Agaricus subrufescens* Peck. *J. Med. Food* 13, 1510-1514.

Harmon, M.E., Sexton, J., Caldwell, B.A., and Carpenter, S.E. (1994). Fungal sporocarp mediated losses of Ca, Fe, K, Mg, Mn, N, P, and Zn from conifer logs in the early stages of decomposition. *Canadian Journal of Forest Research* 24, 1883-1893.

Hedley, M.J., and Stewart, J. (1982). Method to measure microbial phosphate in soils. *Soil Biol. Biochem.* 14, 377-385. doi: 10.1016/0038-0717(82)90009-8.

Jarzyńska, G., Chojnacka, A., Dryżałowska, A., Nnorom, I.C., and Falandysz, J. (2012). Concentrations and bioconcentration factors of minerals in yellow-cracking Bolete (Xerocomus subtomentosus) mushroom collected in Noteć Forest, Poland. *J. Food Sci.* 77, H202-H206. doi: 10.1111/j.1750-3841.2012.02876.x.

Jarzynska, G., Gucia, M., Kojta, A.K., Rezulak, K., and Falandysz, J. (2011). Profile of trace elements in Parasol Mushroom (*Macrolepiota procera*) from Tucholskie Forest. *Journal of Environmental Science and Health, Part B* 46, 741-51. doi: 10.1080/03601234.2011.603986.

Jarzyńska, G., and Falandysz, J. (2012). Trace elements profile of Slate Bolete (*Leccinum duriusculum*) mushroom and associated upper soil horizon. *J. Geochem. Explor.* 121, 69-75. doi: 10.1016/j.gexplo.2012.07.001.

Jayakumar, P., and Tan, T. (2005). Phosphorus solubilization by ectomycorrhizal *Pisolithus tinctorius* in pure culture and in association with *Acacia mangium*. *Symbiosis* 39, 125-130.

Johanson, K.J., Nikolova, I., Taylor, A.F., and Vinichuk, M.M. (2004). "Uptake of elements by fungi in the Forsmark area", in: *SKB*.).

Kojta, A.K., Jarzyńska, G., and Falandysz, J. (2012). Mineral composition and heavy metal accumulation capacity of Bay Bolete (*Xerocomus badius*) fruiting bodies collected near a former gold and copper mining area. *J. Geochem. Explor.* 121, 76-82. doi: 10.1016/j.gexplo.2012.08.004.

Kottke, I., Qian, X.M., Pritsch, K., Haug, I., and Oberwinkler, F. (1998). *Xerocomus badius* - *Picea abies*, an ectomycorrhiza of high activity and element storage capacity in acidic soil. *Mycorrhiza* 7, 267-75. doi: 10.1007/s005720050191.

Koukol, O., Novák, F., and Hrabal, R. (2008). Composition of the organic phosphorus fraction in basidiocarps of saprotrophic and mycorrhizal fungi. *Soil Biology and Biochemistry* 40, 2464-2467. doi: 10.1016/j.soilbio.2008.04.021.

Kovács, D., and Vetter, J. (2015). Chemical composition of the mushroom *Laetiporus sulphureus* (Bull.) Murill. *Acta Aliment. Hung.* 44, 104-110.

Krzystek, L., and Ledakowicz, S. (2000). Stoichiometric analysis of *Kluyveromyces fragilis* growth on lactose. *J. Chem. Technol. Biot.* 75, 1110-1118. doi: 10.1002/1097-4660(200012)75:12<1110::AID-JCTB324>3.3.CO;2-V.

Kułdo, E., Jarzyńska, G., Gucia, M., and Falandysz, J. (2014). Mineral constituents of edible parasol mushroom *Macrolepiota procera* (Scop. ex Fr.) Sing and soils beneath its fruiting bodies collected from a rural forest area. *Chem. Pap.* 68. doi: 10.2478/s11696-013-0477-7.

Lange, H.C., and Heijnen, J.J. (2001). Statistical reconciliation of the elemental and molecular biomass composition of *Saccharomyces cerevisiae*. *Biotechnol. Bioeng.* 75, 334-344. doi: 10.1002/bit.10054.

Larsen, T., Ventura, M., Damgaard, C., Hobbie, E.A., and Krogh, P.H. (2009). Nutrient allocations and metabolism in two collembolans with contrasting reproduction and growth strategies. *Funct. Ecol.* 23, 745-755. doi: 10.1111/j.1365-2435.2009.01564.x.

Larsen, T., Ventura, M., O'Brien, D.M., Magid, J., Lomstein, B.A., and Larsen, J. (2011). Contrasting effects of nitrogen limitation and amino acid imbalance on carbon and nitrogen turnover in three species of Collembola. *Soil Biology and Biochemistry* 43, 749-759. doi: 10.1016/j.soilbio.2010.12.008.

Larsson, C., Vonstockar, U., Marison, I., and Gustafsson, L. (1993). Growth and metabolism of Saccharomyces-cerevisiae in chemostat cultures under carbon-limiting, nitrogen-limiting, or carbon-limiting and nitrogen-limiting conditions. *J. Bacteriol.* 175, 4809-4816. doi.

Lavola, A., Aphalo, P.J., and Lehto, T. (2011). Boron and other elements in sporophores of ectomycorrhizal and saprotrophic fungi. *Mycorrhiza* 21, 155-165. doi: 10.1007/s00572-010-0321-7.

Leach, D., and Gulis, V. (2010). Microbial stoichiometry and homeostasis of nutrient ratios in fungi. *Bridges* 5.

Leick, V. (1968). Ratios between contents of DNA, RNA and protein in different micro-organisms as a function of maximal growth rate. *Nature* 217, 1153-&. doi: 10.1038/2171153a0.

Leite, F.C., Basso, T.O., Pita, W.B., Gombert, A.K., Simoes, D.A., and de Morais, M.J. (2013). Quantitative aerobic physiology of the yeast Dekkera bruxellensis, a major contaminant in bioethanol production plants. *FEMS Yeast Res.* 13, 34-43. doi: 10.1111/1567-1364.12007.

Levi, M.P., and Cowling, E.B. (1969). Role of nitrogen in wood deterioration. VII. Physiological adaptation of wooddestroying and other fungi to substrate deficient in nitrogen. *Phytopathology* 59, 460-468.

Liu, G., Wang, H., Zhou, B., Guo, X., and Hu, X. (2010). Compositional analysis and nutritional studies of Tricholoma matsutake collected from Southwest China. *Journal of Medicinal Plants Research* 4, 1222-1227.

Liu, Y., Sun, J., Luo, Z., Rao, S., Su, Y., Xu, R., and Yang, Y. (2012). Chemical composition of five wild edible mushrooms collected from Southwest China and their antihyperglycemic and antioxidant activity. *Food Chem. Toxicol.* 50, 1238-1244. doi: 10.1016/j.fct.2012.01.023.

Lodge, D.J. (1987). Nutrient concentrations, percentage moisture and density of field-collected fungal mycelia. *Soil Biol. Biochem.* 19, 727-733. doi: 10.1016/0038-0717(87)90055-1.

Low, K.S., and Rogers, P.L. (1984). The macromolecular composition and essential amino acid profiles of strains of *Zymomonas mobilis*. *Appl. Microbiol. Biot.* 19, 75-78.

Lucazechi Sturion, G., and de Camargo Ranzani, M.R. (2000). Mineral composition of edible mushrooms cultivated in Brazil- *Pleurotus* spp and other dehydrated species. *Archivos Latinoamericanos de Nutrición* 50, 102-108.

McMurrough, I., and Rose, A.H. (1967). Effect of growth rate and substrate limitation on the composition and structure of the cell wall of *Saccharomyces cerevisiae*. *Biochem. J.* 105, 189-203.

Merrill, W., and Cowling, E.B. (1966). Role of nitrogen in wood deterioration: amount and distribution of nitrogen in fungi. *Phytopathology* 56, 1083-1090.

Mleczek, M., Siwulski, M., Mikolajczak, P., Gasecka, M., Sobieralski, K., Szymanczyk, M., and Golinski, P. (2015). Content of selected elements in *Boletus badius* fruiting bodies growing in extremely polluted wastes. *Journal of Environmental Science and Health, Part A* 50, 767-75. doi: 10.1080/10934529.2015.1012014.

Mouginot, C., Kawamura, R., Matulich, K.L., Berlemont, R., Allison, S.D., Amend, A.S., and Martiny, A.C. (2014). Elemental stoichiometry of Fungi and Bacteria strains from grassland leaf litter. *Soil Biology and Biochemistry* 76, 278-285. doi: 10.1016/j.soilbio.2014.05.011.

Naguib, M.I., and Salama, A.M. (1966). Effect of colchicine on the mycelial weight and the nitrogen and phosphorus contents of the mycelium of *Cunninghamella* sp. *Can. J. Microbiol.* 12, 91.

Nikkarinen, M., and Mertanen, E. (2004). Impact of geological origin on trace element composition of edible mushrooms. *J. Food Compos. Anal.* 17, 301-310. doi: 10.1016/j.jfca.2004.03.013.

Nissen, T.L., Schulze, U., Nielsen, J., and Villadsen, J. (1997). Flux distributions in anaerobic, glucose-limited continuous cultures of Saccharomyces cerevisiae. *Microbiology+* 143 ( Pt 1), 203-18. doi: 10.1099/00221287-143-1-203.

Nnorom, I.C., Jarzyńska, G., Drewnowska, M., Dryżałowska, A., Kojta, A., Pankavec, S., and Falandysz, J. (2013). Major and trace elements in sclerotium of Pleurotus tuber-regium (Ósū) mushroom—Dietary intake and risk in southeastern Nigeria. *J. Food Compos. Anal.* 29, 73-81. doi: 10.1016/j.jfca.2012.10.001.

Olsson, P.A., Hammer, E.C., Pallon, J., van Aarle, I.M., and Wallander, H. (2011). Elemental composition in vesicles of an arbuscular mycorrhizal fungus, as revealed by PIXE analysis. *Fungal Biol.-UK* 115, 643-648. doi: 10.1016/j.funbio.2011.03.008.

Olsson, P.A., Hammer, E.C., Wallander, H., and Pallon, J. (2008). Phosphorus Availability Influences Elemental Uptake in the Mycorrhizal Fungus Glomus intraradices, as Revealed by Particle-Induced X-Ray Emission Analysis. *Appl. Environ. Microb.* 74, 4144-4148. doi: 10.1128/AEM.00376-08.

Ooijkaas, L.P., Buitelaar, R.M., Tramper, J., and Rinzema, A. (2000). Growth and sporulation stoichiometry and kinetics of Coniothyrium minitans on agar media. *Biotechnol. Bioeng.* 69, 292-300. doi: 10.1002/1097-0290(20000805)69:3<292::AID-BIT7>3.0.CO;2-Z.

Pallon, J., Wallander, H., Hammer, E., Arteaga Marrero, N., Auzelyte, V., Elfman, M., Kristiansson, P., Nilsson, C., Olsson, P.A., and Wegdén, M. (2007). Symbiotic fungi that are essential for plant nutrient uptake investigated with NMP. *Nuclear Instruments and Methods in Physics Research Section B: Beam Interactions with Materials and Atoms* 260, 149-152. doi: 10.1016/j.nimb.2007.02.018.

Parada, G., and Acevedo, F. (1983). On the relation of temperature and RNA content to the specific growth rate in *Saccharomyces cerevisiae*. *Biotechnol. Bioeng.* 25, 2785-2788. doi: 10.1002/bit.260251120.

Parrott, L.M., and Slater, J.H. (1980). THE DNA, RNA and protein-composition of the *Cyanobacterium anacystis-nidulans* grown in light-limited and carbon dioxide-limited chemostats. *Arch. Microbiol.* 127, 53-58. doi: 10.1007/BF00414355.

Ramage, H. (1930). Mushrooms-mineral content. *Nature* 126, 279.

Rezende, L.A., Assis, L.C., and Nahas, E. (2004). Carbon, nitrogen and phosphorus mineralization in two soils amended with distillery yeast. *Bioresource Technol.* 94, 159-167. doi: 10.1016/j.biortech.2003.12.004.

Rosenblitt, A., Agosin, E., Delgado, J., and Perez-Correa, R. (2000). Solid substrate fermentation of Monascus purpureus: Growth, carbon balance, and consistency analysis. *Biotechnol. Progr.* 16, 152-162. doi: 10.1021/bp0000048.

Rudawska, M., and Leski, T. (2005). Macro- and microelement contents in fruiting bodies of wild mushrooms from the Notecka forest in west-central Poland. *Food Chem.* 92, 499-506. doi: 10.1016/j.foodchem.2004.08.017.

Rywińska, A., Juszczyk, P., Wojtatowicz, M., and Rymowicz, W. (2011). Chemostat study of citric acid production from glycerol by Yarrowia lipolytica. *J. Biotechnol.* 152, 54-57. doi: 10.1016/j.jbiotec.2011.01.007.

Schwartzkoff, C.L., and Rogers, P.L. (1982). Glycogen synthesis by glucose-limited *Candida utilis*. *Journal of General Microbiology* 128, 1635-1638.

Singdevsachan, S.K., Patra, J.K., and Thatoi, H. (2013). Nutritional and bioactive potential of two wild edible mushrooms (Lentinus sajor-caju and Lentinus torulosus) from Similipal Biosphere Reserve, India. *Food Sci. Biotechnol.* 22, 137-145. doi: 10.1007/s10068-013-0019-7.

Stark, N. (1972). Nutrient cycling pathways and litter fungi. *BioScience* 22, 355-&. doi: 10.2307/1296341.

Sudheep, N.M., and Sridhar, K.R. (2014). Nutritional composition of two wild mushrooms consumed by the tribals of the Western Ghats of India. *Mycology* 5, 64-72. doi: 10.1080/21501203.2014.917733.

Taylor, A., Fransson, P.M., Hogberg, P., Hogberg, M.N., and Plamboeck, A.H. (2003). Species level patterns in C-13 and N-15 abundance of ectomycorrhizal and saprotrophic fungal sporocarps. *New Phytol.* 159, 757-774. doi: 10.1046/j.1469-8137.2003.00838.x.

Tel, G., Cavdar, H., Deveci, E., Ozturk, M., Duru, M.E., and Turkoglu, A. (2014). Minerals and metals in mushroom species in Anatolia. *Food Additives & Contaminants: Part B* 7, 226-31. doi: 10.1080/19393210.2014.897263.

Tybussek, R., Linz, F., Schugerl, K., Moses, N., Leonard, A.J., and Rouxhet, P.G. (1994). Comparison of the continuous flotation performances of *Saccharomyces cerevisiae* LBG H620 and DSM 2155 strains. *Appl. Microbiol. Biot.* 41, 13-22. doi.

Vaario, L., Pennanen, T., Lu, J., Palmén, J., Stenman, J., Leveinen, J., Kilpeläinen, P., and Kitunen, V. (2015). *Tricholoma matsutake* can absorb and accumulate trace elements directly from rock fragments in the shiro. *Mycorrhiza* 25, 325-334. doi: 10.1007/s00572-014-0615-2.

Vetter, J. (1989). Comparison of mineral elements in Agaricus and Pleurotus fruit bodies. *Zeitschrift für Lebensmittel-Untersuchung und -Forschung* 189, 346-350.

Vetter, J. (1990). Mineral element content of edible and poisonous macrofungi. *Acta Aliment. Hung.* 19, 27-40.

Vetter, J. (1993). Chemical composition of eight edible mushrooms. *Zeitschrift für Lebensmittel-Untersuchung und -Forschung* 196, 224-227.

Vetter, J. (1994). Mineral elements in the important cultivated mushrooms A garicus bisporus and Pleurotus ostreatus. *Food Chem.* 50, 277-279.

Vetter, J. (1995). Mineral and amino-acid contents of edible, cultivated mushroom shiitake (Lentinus-edodes). *Zeitschrift für Lebensmittel-Untersuchung und -Forschung* 201, 17-19.

Vetter, J. (2003). Chemical composition of fresh and conserved Agaricus bisporus mushroom. *Eur. Food Res. Technol.* 217, 10-12. doi: 10.1007/s00217-003-0707-2.

Vetter, J. (2005). Mineral composition of basidiomes of amanita species. *Mycological Research* 109, 746-750.

Vetter, J., Hajdú, C., Gyorfi, J., and Maszlavér, P. (2005). Mineral composition of the cultivated mushrooms Agaricus bisporus, Pleurotus ostreatus and Lentinula edodes. *Acta Aliment. Hung.* 34, 441-451.

Vogt, K.A., Edmonds, R.L., and Grier, C.C. (1981). Biomass and nutrient concentrations of sporocarps produced by mycorrhizal and decomposer fungi in *Abies amabilis* stands. *Oecologia* 50, 170-175. doi: 10.1007/BF00348033.

Vogt, K.A., and Edmonds, R.L. (1980). Biomass and nutrient concentrations of sporocarps produced by mycorrhizal and decomposer fungi in Abies amabilis stands. *Canadian Journal of Botany* 58, 694-698.

Waldron, C., and Lacroute, F. (1975). Effect of growth rate on the amounts of ribosomal and transfer ribonucleic acids in yeast. *J. Bacteriol.* 3, 855-865.

Wang, X., Zhang, J., Li, T., Wang, Y., and Liu, H. (2015). Content and Bioaccumulation of Nine Mineral Elements in Ten Mushroom Species of the GenusBoletus. *J. Anal. Methods Chem.* 2015, 1-7. doi: 10.1155/2015/165412.

Wang, X.M., Zhang, J., Li, T., Li, J.Q., Wang, Y.Z., and Liu, H.G. (2015a). Variations in element levels accumulated in different parts of *Boletus edulis* collected from central Yunnan province, China. *J. Chem.-NY* 2015, 372152.

Wang, X.M., Zhang, J., Li, T., Li, J.Q., Wang, Y.Z., and Liu, H.G. (2015b). ICP-AES determination of mineral content in *Boletus tomentipes* collected from different sites of China. *Spectrosc. Spect. Anal.* 35, 1398-1403.

Washizuka, Y. (1987). Contents of nutrients (N, P, K, Ca, Mg, Na) in mushrooms in forest ecosystems. *Japanese Journal of Ecology* 37, 119-122.

Wehr, C.T., and Parks, L.W. (1969). Macromolecular synthesis in *Saccharomyces cerevisiae* in different growth media. *J. Bacteriol.* 98, 458.

Yildiz, A., Karakaplan, M., and Aydin, F. (1998). Studies on *Pleurotus ostreatus* (Jacq. ex Fr.) Kum. var. *salignus* (Pers. ex Fr.) Konr. et Maubl.: cultivation, proximate composition, organic and mineral composition of carpophores. *Food Chem.* 61, 127-130. doi: 10.1016/S0308-8146(97)00066-6.

Zeng, X., Suwandi, J., Fuller, J., Doronila, A., and Ng, K. (2012). Antioxidant capacity and mineral contents of edible wild Australian mushrooms. *Food Sci. Technol. Int.* 18, 367-379. doi: 10.1177/1082013211427993.

Zhang, D., Zhang, Y., Morawska, E., Bielawski, L., Krasińska, G., Drewnowska, M., Pankavec, S., Szymańska, K., and Falandysz, J. (2013). Tracee elements in *Leccinum scabrum* mushrooms and topsoils from Kłodzka Dale in Sudety Mountains, Poland. *J. Mt. Sci.-Engl.* 10, 621-627. doi: 10.1007/s11629-013-2384-3.

# Appendix S2 Data extracted from literature.

See Appendix S2 file uploaded separately.

# Appendix S3 Data at species level with guild info.

See Appendix S3 file uploaded separately.
